# Potential Influence of Aspirin on Neopterin and Tryptophan Levels in Patients with a Delirium

**DOI:** 10.3390/geriatrics1020010

**Published:** 2016-03-31

**Authors:** Angelique Egberts, Durk Fekkes, Gijsbertus Ziere, Tischa J. M. van der Cammen, Francesco U. S. Mattace-Raso

**Affiliations:** 1 Section of Geriatric Medicine, Department of Internal Medicine, Erasmus University Medical Center, ’s-Gravendijkwal 230, P.O. Box 2040, 3000 CA Rotterdam, The Netherlands; a.egberts@erasmusmc.nl (A.E.); g.ziere@erasmusmc.nl (G.Z.); t.vandercammen@erasmusmc.nl (T.J.M.C.); 2 Department of Clinical Chemistry, Erasmus University Medical Center, ’s-Gravendijkwal 230, P.O. Box 2040, 3000 CA Rotterdam, The Netherlands; d.fekkes@erasmusmc.nl

**Keywords:** delirium, neopterin, tryptophan, NSAIDs, aspirin

## Abstract

In an *in vitro* study, it was found that aspirin might decrease neopterin production and tryptophan degradation. The aim of the present study was to evaluate the possible association between aspirin use and mean neopterin and tryptophan levels in patients with and without a delirium and whether the use of aspirin is associated with a decreased prevalence of delirium. Neopterin and tryptophan levels were determined previously in acutely ill admitted patients aged ≥65 years. The possible influence of aspirin on mean levels of neopterin and tryptophan was investigated with univariate analysis of variance in adjusted models. Eighty-three patients were included; 22 had a delirium. In patients without a delirium (no aspirin (*n* = 31) *versus* aspirin (*n* = 27)), mean neopterin levels were 47.0 nmol/L *versus* 43.6 nmol/L (*p* = 0.645) and tryptophan levels were 33.1 µmol/L *versus* 33.9 µmol/L (*p* = 0.816). In patients with a delirium (no aspirin (*n* = 13) *versus* aspirin (*n* = 9)), mean neopterin levels were 77.8 nmol/L *versus* 71.1 nmol/L (*p* = 0.779) and tryptophan levels were 22.4 µmol/L *versus* 27.3 µmol/L (*p* = 0.439). No difference was found in the distribution of aspirin users between patients with and without a delirium. In this study, we found that the use of aspirin had no significant effect on mean levels of neopterin and tryptophan. However, the raw data suggest that there might be a potential influence in patients with a delirium. Aspirin use was not associated with a decreased prevalence of delirium.

## 1. Introduction

Delirium, an acute neuropsychiatric syndrome, is a common, severe complication in the elderly and is associated with poor clinical outcomes including increased morbidity and mortality, prolonged hospital stay, loss of independence, and increased rates of cognitive decline [1,2]. The pathophysiological mechanisms underlying delirium are still poorly understood, but it is widely accepted that delirium occurs due to a complex interplay among several biochemical pathways. Therefore, it might be required to interrupt in multiple biochemical pathways at the same time to prevent, treat, or to lower the severity of a delirium.

Activation of the immune system, oxidative stress, and disturbances in the serotonergic neurotransmission may all contribute to the development of a delirium. Recently, we found that acutely ill hospitalized elderly patients with a delirium have increased levels of neopterin [3]. Neopterin is produced primarily by activated monocytes and macrophages in response to the pro-inflammatory cytokine interferon-gamma (IFN-γ) and its levels reflect the amount of cell-mediated immune activation and oxidative stress [4,5]. Furthermore, we have found that patients with a delirium have a decreased availability of tryptophan to the central nervous system. This decreased availability might result in a decreased serotonin production in the brain, since tryptophan is the precursor of serotonin [6].

A study of Schroecksnadel *et al*. showed that treatment of stimulated peripheral blood mononuclear cells with aspirin significantly decreased neopterin production and tryptophan degradation *in vitro* [7]. Therefore, the aim of the present study was to evaluate the possible association between aspirin use and mean levels of neopterin and tryptophan in patients with and without a delirium and additionally, whether the use of aspirin is associated with a decreased prevalence of delirium.

## 2. Methods

### 2.1. Participants

The present study was performed within the Delirium In The Old (DITO) study in which mean plasma/serum levels of several biochemical parameters, including neopterin and tryptophan, were compared between patients with and without a delirium [3,6]. In the DITO study, a cross-sectional study, we included patients who were admitted to the wards of Internal Medicine and Geriatrics of the Erasmus University Medical Center and the ward of Geriatrics of the Havenziekenhuis, Rotterdam, The Netherlands. All acutely admitted patients aged ≥65 years were eligible to participate. Exclusion criteria were a diagnosis of Lewy Body dementia, Parkinson’s disease, neuroleptic malignant syndrome, tardive dyskinesia, ongoing treatment with antipsychotics or other psychiatric medications, except haloperidol and benzodiazepines, aphasia, insufficient understanding of the Dutch language, and a Mini Mental State Examination (MMSE) score < 10 points out of 30. Patients with a MMSE < 10 were not included because it can be quite difficult to distinguish between features of severe dementia and delirium at admission, as well as to measure improvement of cognitive function in this group. Additional exclusion criteria for the present study were unclear data regarding the use of non-steroidal anti-inflammatory drugs (NSAIDs) in the days preceding hospital admission as well as the use of aspirin concomitantly with other NSAIDs (as it might be possible that other NSAIDs interfere with aspirin’s potential effect on neopterin and tryptophan levels).

Written informed consent was obtained from all participants. In case of a delirium or cognitive impairment at the time of admission, informed consent was obtained from a representative of the patient. The Medical Ethics Committee of the Erasmus University Medical Center approved the study protocol.

### 2.2. Procedures

All participants were observed daily by the nursing and medical staff and by members of the research team until discharge. To screen for a change in behavior, the 13-item Delirium Observation Screening scale was used during the first five days of admission [8]. The diagnosis of delirium was made by a geriatrician, according to the Diagnostic and Statistical Manual of Mental Disorders, 4th edition (DSM-IV) [9], and was based on the psychiatric examination of the patient, the medical and nursing records, including the Delirium Observation Screening scale scores, and information given by the patient’s closest relative. When the diagnosis of delirium was doubtful, the case was discussed with the geriatric consultation team to gain consensus.

Demographic and clinical data were collected at admission. Age and gender were documented. Cognitive functioning was assessed in absence of a delirium using the MMSE [10]. When it was impossible to score the MMSE during admission because the patient was too ill, the cognitive functioning was discussed with a clinician or assessed with information from the available medical records. When the clinical opinion was that the patient would have a MMSE score ≥ 10, the patient was not excluded from the study. Severity of comorbidities was scored using the Charlson Comorbidity Index. This index encompasses 19 medical conditions and each condition is weighted with a score of 1 to 6 by severity [11]. The physical functionality was assessed using the six-item Katz Activities of Daily Living (ADL) scale and the Barthel Index [12,13]. The instrumental functionality was assessed using the 7-items Older Americans Resource Scale for Instrumental ADL (OARS-IADL) [12]. Frailty was measured with the Identification of Seniors at Risk (ISAR) questionnaire [14]. For all participants the medication at hospital admission was reviewed for the use of NSAIDs (including low dose acetylsalicylic acid and the equivalent drug carbasalate calcium), beta-blockers, diuretics, angiotensin converting enzyme (ACE) inhibitors, angiotensin II receptor antagonists, calcium channel blockers, nitrates, statins, and dipyridamole.

Blood samples of all patients were collected within 48 h after admission. When a patient developed a delirium during the hospital stay, new blood samples were collected within 24 h after the onset of the delirium and were used, instead of the first blood samples, for the statistical analyses.

### 2.3. Biochemical Measurements

Non-fasting blood was collected preferably between 8 and 10 a.m. in an 8-mL tube containing ethylene diamine tetra-acetic acid. After blood sampling, the tubes were protected from light to prevent oxidative loss of neopterin [15], and stored at room temperature to prevent changes in the transfer of amino acids between plasma and blood cells [16]. Within 3 h, the blood was centrifuged for 20 min at 2650 g and 20 °C. The obtained plasma was stored at −80 °C until analysis. 

Plasma neopterin levels were determined by high-performance liquid chromatography after acid oxidation, as previously described [17]. Tryptophan levels were determined by high-performance liquid chromatography with automated pre-column derivatization with ortho-phthalaldehyde [16].

### 2.4. Statistical Analyses

Depending on the distribution of the data, differences in demographic and clinical baseline characteristics between patients with and without a delirium were evaluated using the chi-square test or the Fisher’s exact test for categorical variables and the Mann–Whitney *U*-test or the Student’s *t*-test for continuous variables.

Levels of neopterin and tryptophan were not normally distributed and were, therefore, logarithmically transformed. Univariate one-way analysis of variance was used to investigate the association between mean levels of neopterin and tryptophan (dependent variable) and the use of aspirin in both patients with and without a delirium. For this purpose, analyses were stratified for aspirin use. Age, gender, Charlson Comorbidity Index and statin use were used as covariates. The Charlson Comorbidity Index was added since neopterin levels are found to be increased and tryptophan levels decreased in several medical conditions. Statin use was added since statins might also inhibit neopterin production and tryptophan degradation [18]. The model including neopterin was additionally adjusted for eGFR, since neopterin is excreted mainly by the kidneys [5]. All mean levels and 95% confidence intervals (CI) of neopterin and tryptophan presented in this manuscript are the back-transformed log-values. A two-tailed *p* < 0.05 was defined as statistically significant. All statistical analyses were performed using the Statistical Package for the Social Sciences (SPSS), version 21.0 (IBM Corp., Armonk, NY, USA). GraphPad Prism 5.01 for Windows (GraphPad Software, San Diego, CA, USA) was used to draw the graphs.

## 3. Results

### 3.1. Participant Characteristics

Of the 86 patients enrolled in the DITO study, 80 were included in the stratified analyses to examine the effect of aspirin use on neopterin and tryptophan levels. Three patients were excluded due to unclear data regarding the use of NSAIDs in the days preceding hospital admission, 1 patient used diclofenac and 2 patients used carbasalate calcium concomitantly with another NSAID (diclofenac and etoricoxib respectively). Table 1 represents the baseline characteristics of the included patients. Twenty-two patients were diagnosed with a delirium, of which 21 were admitted to the hospital with a delirium and 1 developed a delirium during admission. No difference was found in the number of aspirin users between patients with and without a delirium (46.6% *versus* 40.9%, *p* = 0.651, respectively).

### 3.2. Analysis of Biochemical Parameters

Mean levels and corresponding 95% CI of neopterin and tryptophan in patients with and without a delirium, stratified for the use of aspirin, are presented in Table 2 and Table 3.

In the group without a delirium, no significant difference was found in the adjusted mean levels of neopterin between patients who used aspirin (43.6 nmol/L, 95% CI: 34.5–55.0) and patients who did not use aspirin (47.0 nmol/L, 95% CI: 37.8–58.3) (*p* = 0.645). Also no difference was found in the adjusted mean levels of tryptophan between patients who used aspirin (33.9 µmol/L, 95% CI: 29.6–38.8) and patients who did not use aspirin (33.1 µmol/L, 95% CI: 29.2–37.6) (*p* = 0.816).

**Figure 1:**
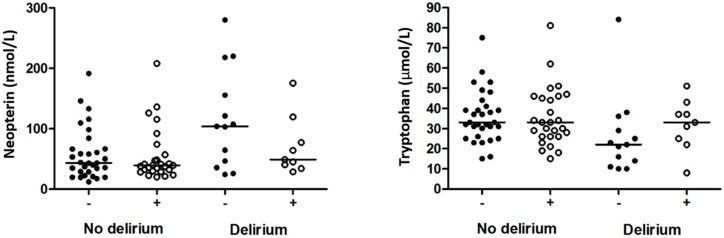
Unadjusted levels of neopterin and tryptophan in patients with and without delirium who used (+) or did not use (−) aspirin. Lines are medians.

In the group with a delirium, unadjusted levels of neopterin seemed to be lower in patients who used aspirin than in those who did not, as shown in Figure 1. However, in this small group the adjusted mean levels of neopterin were not statistically significantly lower in patients who used aspirin (71.1 nmol/L, 95% CI: 43.8–115.6) than in patients who did not use aspirin (77.8 nmol/L, 95% CI: 52.4–115.6) (*p* = 0.779). In addition, unadjusted levels of tryptophan seemed to be higher in patients who used aspirin than in those who did not (Figure 1). However, the adjusted mean levels of tryptophan were not statistically significantly higher in patients who used aspirin (27.3 µmol/L, 95% CI: 18.4–40.5) than in those who did not (22.4 µmol/L, 95% CI: 16.2–30.9) (*p* = 0.439).

## 4. Discussion

In the present study we found that the use of aspirin (exclusively low dose aspirin) was not associated with a decreased prevalence of delirium. Furthermore, we found that mean neopterin and tryptophan levels were not statistically significant affected by the use of aspirin in patients with and without a delirium.

As far as we are aware, this is the first study investigating the possible association between aspirin use and mean neopterin and tryptophan levels in patients with and without a delirium. We found that neopterin and tryptophan levels were not statistically significant affected by the use of aspirin. Therefore, we could not confirm the relationship found *in vitro* by Schroecksnadel *et al.* that aspirin decreases neopterin production as well as tryptophan degradation. These controversial findings may be caused by several factors. First, Schroecksnadel *et al.* found that the inhibition of neopterin production and tryptophan degradation by aspirin was dose-dependent [7]. In our study, all aspirin users used acetylsalicylic acid or carbasalate calcium in a low dose within cardiovascular risk management (80 and 100 mg per day, respectively). It might be possible that these dosages are too low for having a significant effect on neopterin and tryptophan levels. Another possibility is that we did not find an association due to the small sample size. However, in the group with a delirium, neopterin levels seemed to be lower and tryptophan levels seemed to be higher in patients who used aspirin than in those who did not. Therefore, it might be possible that in a larger group these differences would become statistically significant. On the other hand, this trend was not seen in the group without a delirium. Schroecksnadel *et al.* found that aspirin did not influence tryptophan degradation and only minimally affected neopterin production in resting cells [7]. It might be possible that in patients without a delirium neopterin production and tryptophan degradation was not stimulated enough and that we, therefore, did not see a trend in this group.

The potential influence of aspirin on neopterin production and tryptophan degradation in patients with a delirium might be the result of a modulating effect of aspirin on the cytokine IFN-γ. Both the production of neopterin as well as the degradation of tryptophan is IFN-γ dependent. During immune activation, IFN-γ induces in macrophages the enzyme guanosine triphosphate cyclohydrolase-I, which is among others responsible for the production of neopterin [4,5]. IFN-γ also induces the enzyme indoleamine-2,3-dioxygenase which converts tryptophan to kynurenine [19]. In a previous study performed in a chimeric mouse model of giant cell arteritis, aspirin has been demonstrated to be highly effective in suppressing IFN-γ production at doses of 20–100 mg/kg [20]. However, the doses used in that study were much higher than the dose used by our participants and therefore it might be expected that the findings are only generalizable to a lesser extent to the dose used in our study. Interestingly, they also found that another NSAID, indomethacin, was not able to reduce IFN-γ transcription [20]. This might suggest that NSAIDs which are structurally unrelated to aspirin are not able to affect neopterin and tryptophan levels. In line with this hypothesis, Forrest *et al.* found in patients with osteoporosis after two years of drug treatment that additional pain treatment with a NSAID did not decrease neopterin levels and did not increase tryptophan levels in comparison with patients who did not use NSAIDs [21]. The authors note that patients taking NSAIDs might be among the more severely affected patients in whom disease control could be difficult and this could have influenced their results [21].

Furthermore, we found that the use of aspirin was neither associated with a decreased nor with an increased prevalence of delirium, despite it has been speculated that NSAIDs increase the risk of a delirium. In a systematic review it was found that research on the association of NSAIDs with delirium is limited and that the association remains uncertain [22]. It is important to note that in the present study the association between the use of low dose aspirin and delirium was evaluated. Therefore, it might be still possible that other NSAIDs are associated with an increased risk of a delirium.

### Limitations and Strengths

This study has some limitations. First, the cross-sectional design limits the ability to identify a causal relationship between aspirin use, neopterin and tryptophan levels, and the prevalence of delirium. Therefore, the results of this study should be considered as hypothesis generating. Second, the relatively small sample size decreased the ability to detect a possible association between aspirin use and mean neopterin and tryptophan levels in patients with a delirium. Third, delirium severity was not scored in our study. It might be possible that the use of aspirin does not prevent delirium, but that it will decrease delirium severity (as aspirin really inhibits neopterin production and tryptophan degradation as it seems). Fourth, tryptophan levels could be influenced by dietary intake. In this study, we were not able to adjust for dietary status and this might have influenced our results. However, since possible food intake was random and blood was collected between 8 and 10 a.m., we think that our results are only minimally influenced by this. Finally, we were not able to evaluate whether the use of NSAIDs other than aspirin will have a potential influence on neopterin and tryptophan levels. Since other NSAIDs, used to treat inflammation and pain, are only limited prescribed to elderly patients due to their negative effects on renal function, it would probably only be interesting to investigate this for diseases in a younger population in which neopterin and tryptophan are also involved and not for delirium in elderly patients.

The present study has several strengths. First, the intensive monitoring of clinical symptoms of patients with a delirium until discharge and the DSM-IV diagnosis by a geriatrician makes it less likely that we missed a delirium or misdiagnosed symptoms. Second, we have performed statistical analyses in a relatively homogeneous group of patients, since all of them used low dose aspirin.

## 5. Conclusions

In this study in older, acutely ill hospitalized patients, we did not find a statistically significant effect of aspirin use on neopterin and tryptophan levels in patients with and without a delirium. However, in patients with a delirium, neopterin levels seemed to be lower and tryptophan levels seemed to be higher in patients who used aspirin compared with those who did not. Larger studies might be needed to investigate this potential influence of aspirin use on neopterin production and tryptophan degradation in patients with a delirium.

## Figures and Tables

**Table 1:** Demographic and clinical baseline characteristics of the study participants.

Variable	No Delirium (*n* = 58)	Delirium (*n* = 22)	*p*-Value
Gender male	28 (48.3)	9 (40.9)	0.555 *
Age in years	80.4 ± 7.5	85.8 ± 4.1	0.002 ^‡^
MMSE ^||^	25.0 (22.0–28.0)	20.0 (17.3–24.3)	0.000 ^†^
Katz ADL score ^¶^	0.0 (0.0–3.0)	3.5 (1.0–11.3)	0.013 ^†^
OARS-IADL score ^#^	5.0 (0.0–10.0)	10.0 (3.0–14.0)	0.037 ^†^
Barthel Index **	18.0 (13.0–20.0)	16.0 (9.0–19.0)	0.050 ^†^
ISAR score ^††^	4.0 (2.5–6.0)	6.0 (5.0–7.0)	0.000 ^†^
Charlson Comorbidity Index ^‡‡^	2.00 (1.00–3.00)	2.00 (1.00–3.25)	0.202 ^†^
eGFR (mL/min)	64.3 ± 25.3	48.0 ± 24.7	0.011 ^‡^
Aspirin at admission	27 (46.6)	9 (40.9)	0.651 *
Type of aspirin:			
Acetylsalicylic acid	12 (44.4)	5 (55.6)	
Carbasalate calcium	15 (55.6)	4 (44.4)	
Beta-blockers	17 (29.3)	6 (27.3)	0.857 *
Diuretics	22 (37.9)	7 (31.8)	0.612 *
ACE inhibitors	14 (24.1)	6 (27.3)	0.772 *
Angiotensin II receptor antagonists	8 (13.8)	2 (9.1)	0.719 ^§^
Calcium channel blockers	13 (22.4)	3 (13.6)	0.536 ^§^
Nitrates	5 (8.6)	1 (4.5)	1.000 ^§^
Statins	27 (46.6)	3 (13.6)	0.007 *
Dipyridamole	5 (8.6)	0 (0.0)	0.315 ^§^

Values are expressed as mean ± SD for normally distributed continuous variables, median (interquartile range) for not normally distributed continuous variables and *n* (percentages) for categorical variables. * Chi-square test. ^†^ Mann–Whitney *U*-test; ^‡^ Student’s *t*-test; ^§^ Fisher’s exact test; ^||^ range 0 (severe cognitive impairment) to 30 (no cognitive impairment); ^¶^ range 0 (no disability) to 12 (severe disability); ^#^ range 0 (no disability) to 14 (severe disability); ** range 0 (severe disability) to 20 (no disability); ^††^ scores ≥ 2 indicate a high risk for functional decline; ^‡‡^ range 0 to 37 (severe burden of comorbidities).

**Table 2:** Neopterin levels (nmol/L) in patients with and without a delirium, stratified for the use of aspirin.

No Delirium	No Aspirin (*n* = 31)	Aspirin (*n* = 27)	*p*-Value
Model 1	45.8 (36.2–57.9)	44.9 (34.9–57.8)	0.908
Model 2	47.0 (37.8–58.3)	43.6 (34.5–55.0)	0.645
**Delirium**	**No Aspirin (*n* = 13)**	**Aspirin (*n* = 9)**	***p*-Value**
Model 1	88.3 (57.7–135.2)	59.3 (35.6–98.9)	0.228
Model 2	77.8 (52.4–115.6)	71.1 (43.8–115.6)	0.779

Values are expressed as mean (95% CI) and are the back-transformed log_10_ values. Model 1: not adjusted. Model 2: adjusted for age, gender, Charlson Comorbidity Index, eGFR, statin use.

**Table 3:** Tryptophan levels (µmol/L) in patients with and without a delirium, stratified for the use of aspirin.

No Delirium	No Aspirin (*n* = 31)	Aspirin (*n* = 27)	*p*-Value
Model 1	33.8 (29.6–38.6)	33.1 (28.7–38.2)	0.835
Model 2	33.1 (29.2–37.6)	33.9 (29.6–38.8)	0.816
**Delirium**	**No Aspirin (*n* = 13)**	**Aspirin (*n* = 9)**	***p*-Value**
Model 1	21.6 (15.4–30.3)	28.8 (19.2–43.2)	0.269
Model 2	22.4 (16.2–30.9)	27.3 (18.4–40.5)	0.439

Values are expressed as mean (95% CI) and are the back-transformed log_10_ values. Model 1: not adjusted. Model 2: adjusted for age, gender, Charlson Comorbidity Index and statin use.
